# An Investigation of Differential Item Functioning in the Infection Control Contact Isolation Precaution Instrument Among Nurses and Physicians

**DOI:** 10.1155/bmri/9324434

**Published:** 2026-09-16

**Authors:** Nahid Khoddami, Zahra Shayan, Peyman Jafari, Mehrdad Askarian

**Affiliations:** ^1^ Department of Biostatistics, School of Medicine, Shiraz University of Medical Sciences, Shiraz, Iran, sums.ac.ir; ^2^ Departments of Biostatistics and Epidemiology, School of Public Health, Shahid Sadoughi University of Medical Sciences, Yazd, Iran, ssu.ac.ir; ^3^ Department of Biostatistics, Trauma Research Center, School of Medicine, Shiraz University of Medical Sciences, Shiraz, Iran, sums.ac.ir; ^4^ Department of Community Medicine, Medicinal and Natural Products Chemistry Research Center, Shiraz University of Medical Sciences, Shiraz, Iran, sums.ac.ir

**Keywords:** contact isolation precautions, differential item functioning, nurse, physician

## Abstract

**Objective:**

Healthcare workers must understand disease transmission and adhere to appropriate precautions to prevent infections. However, differences in how questionnaire items are interpreted may lead to misleading comparisons between groups of healthcare professionals. This study examines differential item functioning (DIF) in the contact isolation precautions questionnaire between nurses and physicians.

**Methods:**

In this cross‐sectional study, 676 nurses and 138 physicians completed the questionnaire in May 2019. DIF was assessed using Firth binary logistic regression.

**Results:**

Among the 814 participants, 83% were nurses and 17% were physicians. DIF analysis identified item 8 in the knowledge domain as significant. After its removal, mean scores between the two groups remained significantly different (*p* < 0.001). In the attitude domain, removing Items 6 and 8 eliminated significant score differences. However, in the practice domain, mean score differences persisted even after removing Items 2, 3, and 6 individually or together (*p* < 0.001). Nurses consistently scored higher in all domains.

**Conclusion:**

Nurses and physicians may interpret some items of the contact isolation precautions questionnaire differently, which may affect comparisons of knowledge, attitude, and practice scores. Therefore, measurement equivalence should be considered when comparing these groups. These findings are specific to the questionnaire and study population examined and should not be generalized to other infection prevention and control instruments or populations. Further multicenter studies are warranted.

**Implications for Nursing Practice:**

Differences in item functioning should be considered when comparing nurses and physicians using this questionnaire. Despite relatively high knowledge and attitude scores, practice scores remained low, particularly among physicians, highlighting the importance of considering measurement equivalence when interpreting these differences and planning educational strategies.

## 1. Background

Healthcare‐associated infections (HAIs) affect over 1.4 million people, with a higher incidence in developing countries [1–3]. Healthcare workers (HCWs) face a significant risk of HAIs due to exposure to patients′ bodily fluids, blood, and various infectious pathogens during clinical practice [4, 5]. Among HCWs, nurses and physicians are most commonly exposed, accounting for 41% and 31%, respectively. Preventing the transmission of multidrug‐resistant organisms (MDROs) is a major challenge for the healthcare system due to the increasing prevalence of infectious diseases such as Hepatitis B, C, HIV, and emerging infectious diseases [6–9].

Different strategies have been proposed to limit the spread of MDROs, emphasizing prompt patient identification and effective containment measures [10, 11]. The Centers for Disease Control and Prevention (CDC) has issued guidelines for hospital isolation precautions, crucial for HCWs caring for patients [12].

Contact precautions are vital to MDRO prevention, encompassing practices such as isolating patients in private rooms, maintaining hand hygiene before and after patient contact, and using gowns and gloves when interacting with patients or their environment [13]. These precautions are essential for protecting patients and HCWs from infectious agents and highlight the importance of adequate knowledge and experience regarding disease transmission and infection prevention measures among healthcare professionals, including medical students, nurses, and physicians [13].

An important strategy for protecting HCWs and preventing occupational injuries is to provide appropriate training and strengthen their knowledge [13]. Adequate knowledge and proper practices are crucial for controlling nosocomial infections and minimizing the risk to HCWs [14, 15].

Assessing the knowledge, attitude, and practice (KAP) of HCWs is typically done using standard or custom questionnaires. A reliable questionnaire is essential as it ensures consistency and accuracy in the assessment process, enabling effective interventions to improve patient safety and prevent HAIs. Previous studies have evaluated training effectiveness on isolation precautions by comparing mean scores among different groups [16–18]. However, a significant limitation in these studies is that they compared KAP scores at the scale level rather than the item level of the questionnaires. Therefore, before comparing KAP scores across subgroups of HCWs, it is essential to establish measurement invariance.

Establishing measurement equivalence is a critical aspect of instrument development and validation. It ensures that questionnaire scores remain unbiased and reflect the same construct for all respondents, regardless of factors such as gender, race, or ethnicity.

Measurement invariance can be examined using differential item functioning (DIF) analysis, which evaluates whether individuals from different groups respond differently to specific items after accounting for differences in their overall level of the measured construct. DIF indicates potential bias, and researchers can address it by modifying or removing biased items to improve questionnaire validity and reliability.

Identifying and addressing DIF is essential for ensuring fairness, validity, and accuracy in questionnaires or tests, as it helps detect hidden biases [19, 20].

Unlike simple comparisons of total scores between groups, item‐level analysis can identify where group differences occur and whether systematic differences in item functioning are present [20]. In KAP investigations, item equivalence has not been assessed [16–18, 21]. Consequently, it remains unclear whether the observed differences in total scores among different groups, such as nurses and physicians, reflect genuine disparities in their understanding of the tested concepts or if they are influenced by items exhibiting bias for or against certain groups.

Measurement equivalence of contact precautions questionnaire items among nurses and physicians has received relatively little attention, particularly when assessed using Firth logistic regression. The Firth logistic regression enhances parameter estimate accuracy, particularly in small sample sizes or when dealing with rare responses in questionnaire items [22]. Mousavi et al. [23] recently assessed DIF among medical students and nurses in a standard isolation precaution questionnaire. This study is aimed toat identifying items with substantive DIFdifferential item functioning in the KAPknowledge, attitude, and practice domains using Firth logistic regression. Subsequently, it will compare total scores between nurses and physicians, both with and without DIF items. This study is the first of its kind in this area.

## 2. Methods

### 2.1. Design and Study Population

This cross‐sectional study included 676 nurses who had completed their nursing education and were working at the study hospital in May 2019, and 138 physicians who had completed their 7‐year medical education and were working at Namazi Hospital in Shiraz, Iran. We made sure that nobody had filled out the surveys more than once throughout that period, and there was no rotation.

Five trained interviewers explained the study objectives to the participants and assured them that their responses would remain anonymous. The interviewers gave the questionnaire to individuals who agreed to participate after getting their spoken informed consent. People who refused to take part or filled out questionnaires incompletely were not included.

### 2.2. Contact Isolation Precautions Questionnaire

The questionnaire collected demographic information and assessed knowledge, attitudes, and practices related to contact precautions. It was previously evaluated by four experts from the Iranian Association of Nosocomial Infection Control [16–18]. Eight facets of KAP were covered by these questions (Table 1).

**Table 1:** The information of demographics across nurses and physicians.

	Physicians (*n* = 138)	Nurses (*n* = 676)	*p*
Sex			
Male	85 (61.6)	87 (12.9)	*p* < 0.001
Female	53 (38.4)	589 (87.1)	
Previously attended a training course regarding contact isolation precautions			
No	104 (75.4)	181 (26.8)	*p* < 0.001
Yes	34 (24.6)	495 (73.2)	
Age (mean ± SD)	33.7 ± 5.0	29.7 ± 6.2	*p* < 0.001

Abbreviation: SD, standard deviation.

Correct answers received a score of 1 in the knowledge domain, whereas other answers received a 0. Notably, Item 8 in the knowledge domain (“Double plastic gloving”) was reverse‐scored: “Incorrect” was coded as 1 (correct) and “Correct” as 0, consistent with the original instrument design to minimize response bias.

Practice items used a five‐point Likert scale (*always* = 5, *often* = 4, *sometimes* = 3, *rarely* = 2, and *never* = 1). Responses of “always” and “often” were coded as good performance (1), whereas other choices indicated weak performance (0). Attitude choices ranged from “very high” to “unimportant.” “Very high” and “high” were considered a positive attitude (1), whereas the other choices indicated a negative attitude (0). Thus, each domain had a minimum score of 0 and a maximum score of 8. In this study, the reliability index using Cronbach′s alpha was 0.60, 0.74, and 0.81 for the KAP domains, respectively.

### 2.3. Statistical Analysis

We used Firth binary logistic regression to assess measurement equivalence at the item level. Given the expected imbalance in sample sizes between nurses (*n* = 676) and physicians (*n* = 138), Firth logistic regression was chosen to provide unbiased parameter estimates and maintain valid inference in the presence of unequal group sizes [22, 24, 25]. Comparative simulation studies have also confirmed the advantage of Firth′s method for DIF detection under imbalanced conditions [26].

Firth logistic regression is useful in the presence of data separation or quasiseparation, which can lead to biased and unstable estimates in standard logistic regression. It introduces a penalization term, Firth′s bias‐reducing penalized likelihood, to reduce bias. This approach is especially beneficial for small sample sizes or when dealing with rare responses in a questionnaire [22].

All eight items in each of the three domains were analyzed as dependent variables in separate binary logistic regression models. By comparing various models, DIF, both uniform and nonuniform, was identified using the LR, adjusted for sex, age, and training course:

(1) logitπ=lnpYi=11−pYi=1=α+β1×sex+β2×age+β3×training+β4×θ,

(2) logitπ=lnpYi=11−pYi=1=α+β1×sex+β2×age+β3×training+β4×θ+β5×G,

(3) logitπ=lnpYi=11−pYi=1=α+β1×sex+β2×age+β3×training+β4×θ+β5×G+β6×θ×G.

In the models, “G” represents the variable related to group membership, whereas “*θ*” signifies the level of ability, the observed sum score in each domain. This ability level is calculated by summing the items within a domain, and “*π*” represents the probability of a correct answer to a specific item.

DIF was assessed using differences in −2 log‐likelihood. The authors compared the [−2 log‐likelihood] of Models (1) and (3), both of which follow a Chi‐square distribution with two degrees of freedom, to identify DIF. For investigating uniform DIF (U‐DIF), the authors compared the [−2 log‐likelihood] of Models (1) and (2), which follow a chi‐square distribution with one degree of freedom. This test examines whether differences between groups in the probability of specific item responses remain constant at all ability levels.

To test nonuniform DIF (NU‐DIF), the authors compared the [−2 log‐likelihood] of Models (2) and (3), also with a chi‐square distribution with one degree of freedom. This test assesses whether there is an interaction between group membership and individual ability [22].

To assess the practical or clinical importance of the identified DIF, we used the criteria proposed by Zumbo and Gelin (ZG) and by Crane, van Belle, and Larson (CvBL). According to the ZG standard, DIF values below 0.035 are considered insignificant, those between 0.035 and 0.07 are considered moderate, and values above 0.07 are deemed significant. In line with the CvBL criteria, any changes exceeding 10% in the coefficients of Models (1) and (2) are regarded as a significant impact on the diagnosis of DIF [27–29].

We used curves to assess the magnitude and direction of DIF between nurses and physicians, providing a clearer understanding of DIF detection. These curves were presented exclusively for items exhibiting U‐DIF, as items with NU‐DIF tend to offset the DIF effect at the domain level.

Statistical analyses were conducted using SPSS Version 18.0 and the logistf package in R (Version ≥ 3.0.0) [24, 25]. The *p* value < 0.05 was considered statistically significant.

## 3. Results

### 3.1. Participants

The study included 814 participants, comprising 676 nurses (83%) and 138 physicians (17%). Among nurses, 87.1% were females. Among physicians, 38.4% were females. This gender distribution showed a statistically significant difference (*p* < 0.001). The mean age of nurses was 29.7 ± 6.2 years; for physicians, it was 33.7 ± 5.0 years. Additionally, 73.2% of nurses and 24.6% of physicians had attended training courses on isolation precautions (Table 1).

The items of the contact precautions questionnaire are displayed in Table 2. These results indicated that 59.0%–95.1% of nurses and 44.2%–100% of physicians answered the knowledge items correctly.

**Table 2:** Frequency distribution of answers regarding knowledge, attitude, and practice on contact isolation precautions across nurses and physicians.

Item	Contact precaution items	Correct knowledge %		Positive attitude %		Practice compliant %	
		Nurses	Physicians	Nurses	Physicians	Nurses	Physicians
1	Isolation of patients needing contact precautions in a private room.	84.8	73.9	90.7	74.6	87.1	29.0
2	Gloving on entry and removing gloves before leaving patient′s room.	95.1	100.0	92.6	95.7	90.1	4.3
3	Washing hands with antibacterial agent on removal of gloves.	89.2	87.7	90.8	83.3	88.0	55.1
4	Wearing gown on entry to patient′s room.	93.8	96.4	90.2	94.9	88.5	10.1
5	Notifying ward prior to receiving patient.	89.1	100.0	89.1	97.8	85.8	2.2
6	Dedicating noncritical patient care equipment to isolated patient.	89.6	44.2	89.6	52.9	85.7	73.9
7	Cleaning and disinfecting all common equipment between isolated patients.	87.4	87.0	87.9	84.8	83.1	22.5
8	Double plastic gloving for prevention of transmission of hospital‐acquired infections.	59.0	97.1	26.2	3.6	41.4	4.3

With the exception of Item 8 (use of double gloves), the percentage of positive responses among nurses (87.9%–92.6%) was higher than that among physicians (52.9%–97.8%) for most attitude items. Although the performance of nurses (83.1%–90.1%) was better than physicians (2.2%–73.9%) in all items except Item 8, the performance of nurses and physicians was relatively low (41.4% vs. 4.3%).

### 3.2. Results of DIF

The response rate was 85.7% (814/950). Firth logistic regression results for detecting DIF across nurses and physicians, adjusted for age, sex, and training course, are presented in Table 3.

**Table 3:** The results of DIF analysis across nurses and physicians based on PML methods.

Items	DIF		Uniform DIF					Nonuniform DIF			
	*χ* ^2^	*p*	*β*_5_^a^ (SE^b^)	*χ* ^2^	*p*	*Δ*R^2c^	CVBL	*β*_6_^a^ (SE)	*χ* ^2^	*p*	*Δ*R^2^
Knowledge											
1	**25.203**	**< 0.001**	**1.133 (0.318)**	12.257	< 0.001	0.017	2.207	**−1.222 (0.397)**	**12.946**	**< 0.001**	**0.019**
2	2.478	0.289	−2.671 (1.516)	4.219	0.039	0.019	6.377	0.301 (0.943)	0.000	1.000	< 0.001
3	0.000	1.000	0.267 (0.402)	0.139	0.708	0.001	0.122	0.023 (0.333)	0.000	1.000	0.001
4	0.872	0.646	0.889 (0.615)	1.123	0.289	0.005	3.481	0.269 (0.471)	0.000	1.000	0.002
5	**11.713**	**0.002**	**−3.398 (1.440)**	**14.284**	**< 0.001**	**0.031**	**1.935**	0.759 (0.992)	0.000	1.000	< 0.001
6	**123.458**	**< 0.001**	**3.535 (0.374)**	113.277	< 0.001	0.153	16.974	**−1.094 (0.375)**	**10.181**	**0.001**	**0.015**
7	**7.623**	**0.022**	**0.139 (0.381)**	0.000	1.000	0.001	0.174	**−1.417 (0.628)**	**7.765**	**0.005**	**0.014**
8	**93.253**	**< 0.001**	**−3.681 (0.538)**	**93.031**	**< 0.001**	**0.091**	**14.436**	−0.749 (0.572)	0.221	0.637	0.001
Attitude											
1	**12.683**	**0.001**	**1.258 (0.348)**	**12.668**	**< 0.001**	**0.023**	**2.229**	−0.080 (0.262)	0.014	0.904	0.001
2	2.359	0.307	−0.844 (0.582)	1.828	0.176	0.006	1.693	−0.295 (0.387)	0.530	0.466	0.004
3	4.281	0.117	0.673 (0.388)	2.676	0.101	0.006	0.863	−0.432 (0.339)	1.605	0.205	0.005
4	**17.987**	**< 0.001**	**−1.854 (0.579)**	11.384	< 0.001	0.026	0.165	**0.786 (0.283)**	**6.602**	**0.010**	**0.017**
5	**13.015**	**0.001**	**−2.476 (0.786)**	**13.676**	**< 0.001**	**0.030**	**0.075**	0.271 (0.395)	0.000	1.000	0.001
6	**38.541**	**< 0.001**	**2.055 (0.356)**	**35.043**	**< 0.001**	**0.051**	**0.560**	−0.725 (0.377)	3.497	0.061	0.007
7	**11.148**	**0.003**	**0.070 (0.386)**	0.000	1.000	0.001	0.248	**−1.694 (0.630)**	**11.405**	**< 0.001**	**0.021**
8	**19.283**	**< 0.001**	**1.827 (0.478)**	**20.239**	**< 0.001**	**0.024**	**15.751**	0.478 (0.366)	0.000	1.000	< 0.001
Practice											
1	3.694	0.157	−0.907 (0.427)	4.377	0.036	0.006	11.508	−0.084 (0.255)	0.000	1.000	0.001
2	**15.754**	**< 0.001**	**2.302 (0.634)**	**13.842**	**< 0.001**	**0.017**	**11.362**	−1.400 (0.961)	1.912	0.166	0.005
3	**9.676**	**0.007**	**−1.128 (0.389)**	**9.151**	**0.002**	**0.013**	**14.608**	0.202 (0.203)	0.525	0.468	0.002
4	0.000	1.000	0.117 (0.511)	0.000	1.000	< 0.001	1.494	0.053 (0.307)	0.000	1.000	0.001
5	**40.812**	**< 0.001**	2.969 (0.649)	28.048	< 0.001	0.031	15.061	**1.906 (0.581)**	**12.764**	**< 0.001**	**0.016**
6	**109.850**	**< 0.001**	**−5.249 (0.640)**	**111.010**	**< 0.001**	**0.160**	**95.728**	0.006 (0.334)	0.000	1.000	< 0.001
7	**33.177**	**< 0.001**	−2.104 (0.535)	16.800	< 0.001	0.019	27.191	**3.422 (1.664)**	**16.376**	**< 0.001**	**0.020**
8	**22.105**	**< 0.001**	1.160 (0.490)	5.482	0.019	0.005	22.793	**−1.284 (0.409)**	**16.622**	**< 0.001**	**0.016**

^a^Coefficients in Formulas 2 and 3.

^b^SE, standard error.

^c^ *Δ*R^2^ is the ZG criterion.

In the knowledge domain, two out of eight items (25%)—specifically Items 5 and 8—displayed U‐DIF, whereas three items (38%)—items 1, 6, and 7—displayed NU‐DIF. According to CvBL and ZG criteria, Item 8 exhibited considerable DIF across nurses and physicians.

In the attitude domain, six out of eight items (75%) showed significant DIF. Among these, four items (50%)—Items 1, 5, 6, and 8—displayed U‐DIF, whereas two items (25%)—Items 4 and 7—displayed NU‐DIF. According to the CvBL criterion, Item 8 had considerable DIF across nurses and physicians. Based on the ZG criterion, Item 6 also exhibited moderate DIF.

In the practice domain, six out of eight items (75%) showed significant DIF. Of these, three items (38%)—Items 2, 3, and 6—displayed U‐DIF, whereas three items (38%)—Items 5, 7, and 8—displayed NU‐DIF. According to the CvBL criterion, Items 2, 3, and 6 exhibited considerable DIF across nurses and physicians. Based on the ZG criterion, Item 6 also exhibited a large DIF.

**Figure 1:**
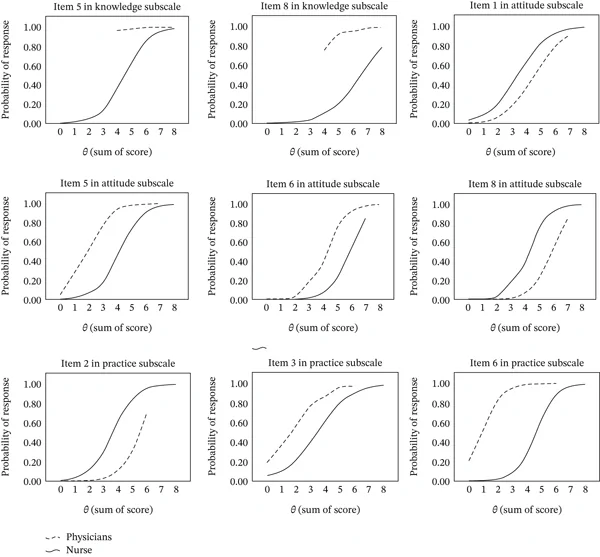
Probability values for items with iniform DIF in knowledge, attitude, and practice among nurses and physicians.

Figure 1 illustrates the probability of answering items among nurses and physicians. The figure shows that two items—Items 5 and 8—exhibited U‐DIF in one direction in the knowledge domain, and their effects could not be canceled out for this domain.

Additionally, in the attitude domain, Items 1, 6, and 8 exhibited U‐DIF in one direction, wherea Item 5 showed DIF in the opposite direction. Consequently, their effects did not cancel each other out at the domain level. A similar pattern was observed in the practice domain, where Items 3 and 6 displayed DIF in one direction. In contrast, Item 2 exhibited DIF in the opposite direction, indicating that their effects could not be canceled in this domain.

### 3.3. Impact of DIF on Mean Comparison

To assess the potential impact of items with significant DIF on score comparisons between nurses and physicians, items with U‐DIF were removed from the domains. Table 4 presents the results:

• Knowledge score: There was no statistically significant difference between nurses and physicians (*p* = 0.890). However, after removing Item 8 (which had a substantial effect), mean scores differed significantly between the two groups (*p* < 0.001).
• Attitude score: A statistically significant difference was observed between nurses and physicians (*p* < 0.001), with nurses having significantly higher scores. After removing Items 6 and 8 from the attitude domain, the mean score between nurses and physicians was no longer statistically significant (*p* = 0.375).
• Practice score: A statistically significant difference was noted between nurses and physicians (*p* < 0.001), with nurses having higher scores. Removing Items 2, 3, and 6 individually, as well as all three items simultaneously, did not eliminate the significant mean score difference between the two groups.

**Table 4:** Comparison of the mean score between nurses and physicians before and after the elimination of items with DIF.

	Physicians (*n* = 138) *m* *e* *a* *n* ± *S* *D* ^a^	Nurses (*n* = 676) *m* *e* *a* *n* ± *S* *D*	*p*	Cohen′s effect size
Scores without eliminating DIF items				
Knowledge	6.86 ± 0.98	6.88 ± 1.41	0.889	0.02
Attitude	5.88 ± 1.13	6.57 ± 1.49	< 0.001	0.52
Practice	2.01 ± 1.30	6.50 ± 1.73	< 0.001	2.93
Scores with eliminating DIF items				
Knowledge (delete Item 8)	5.89 ± 0.95	6.30 ± 1.31	< 0.001	0.36
Attitude (delete Items 6 and 8)	5.31 ± 0.96	5.41 ± 1.27	0.375	0.09
Practice (delete Items 2, 3, and 6)	0.68 ± 1.00	3.86 ± 1.10	< 0.001	3.03

^a^SD, standard deviation.

## 4. Discussion

This is the first study to assess the measurement equivalence of the contact isolation precautions questionnaire between nurses and physicians. Importantly, detecting DIF in one or more items does not necessarily mean that scores are incomparable across groups. Once DIF is identified, its severity and direction, as well as its clinical and practical relevance, should be evaluated.

A relatively high proportion of items exhibited statistically significant DIF across the KAP domains, suggesting that the instrument may not function equivalently for nurses and physicians. However, only a subset of these items showed DIF of sufficient magnitude to meaningfully influence score comparisons. Specifically, one item in the knowledge domain, two in the attitude domain, and three in the practice domain demonstrated practically important DIF according to the predefined classification criteria. Thus, statistical significance alone should not be interpreted as evidence that all affected items have a meaningful impact on comparisons between nurses and physicians. This pattern is unlikely to reflect coding errors or model instability. In the present study, item coding—including the reverse‐scored knowledge item—followed the original instrument design, and Firth logistic regression was used to reduce estimation bias under conditions of unequal group sizes and sparse responses. A more plausible explanation is that nurses and physicians interpret certain items differently because of differences in professional education, clinical roles, and routine exposure to infection‐control practices.

The observed pattern also has important implications for the interpretation of questionnaire scores. Although statistically significant DIF does not necessarily invalidate the instrument, it indicates that some observed differences between nurses and physicians may reflect differences in item functioning rather than true differences in the underlying constructs. Therefore, direct comparisons based solely on total scores should be interpreted with caution. At the same time, because only a subset of items demonstrated practically important DIF, the findings do not suggest that the questionnaire is fundamentally flawed. Rather, refinement of specific items exhibiting substantial DIF may improve cross‐professional comparability while preserving the overall structure of the instrument.

In the knowledge domain, DIF was detected in 63% of items, with Item 8 being the only item showing practically important DIF. Removing Item 8 significantly affected the mean scores: The initially nonsignificant difference between nurses and physicians became significant after its removal. This indicates that the two professional groups may have perceived or interpreted this item differently. Furthermore, the observed internal consistency (Cronbach′s alpha = 0.60) in this domain reflects the inherent heterogeneity of knowledge items, as distinct clinical facts may not be highly correlated. The presence of DIF in Item 8 may also introduce measurement noise, contributing to the moderate alpha level in this subscale [30].

A similar pattern was observed in the attitude domain, where 75% of items exhibited DIF, with 33% (two of six) of the DIF items showing substantial DIF. After excluding Items 6 and 8, which exhibited the most substantial DIF according to the predefined criteria, the mean score difference between nurses and physicians was no longer statistically significant. This finding suggests that the two groups may respond differently to certain items in the contact precautions questionnaire, with nurses tending to report slightly more positive attitudes.

Item 8, “Wearing two plastic gloves simultaneously is equal to one surgical glove for taking care of patients?,” exhibited influential DIF in both the knowledge and attitude domains, mainly because of its reverse‐scored format in the knowledge domain. Unlike the other knowledge items, for which “Correct” received a score of 1, “Incorrect” was coded as 1 (correct) for this item, consistent with the original instrument design to minimize response bias. The change in mean scores before and after its removal indicates that this item functioned differently across the two professional groups.

Beyond its reverse‐scored format, the content of this item may also have contributed to its differential functioning. Unlike most knowledge items, which assess general recommendations for contact isolation, this item requires respondents to judge whether wearing two plastic gloves is equivalent to wearing a surgical glove. Rather than simply recalling an established recommendation, respondents must interpret a specific procedural statement, which may be influenced by differences in professional education, clinical responsibilities, and routine exposure to infection‐control practices. Consequently, nurses and physicians may not interpret the item in the same way. Although the exact mechanism underlying the observed DIF cannot be determined from the present study, these findings suggest that both the wording and content of technically specific items should be carefully evaluated when developing or adapting instruments for comparisons across different healthcare professional groups.

In the practice domain, DIF was observed in 75% of items, with 50% (three of six) of the DIF items exhibiting substantial DIF. Because removing a large number of DIF items may compromise score validity [31], items with substantial DIF were evaluated through sensitivity analyses. Unlike the attitude domain, where the between‐group difference disappeared after removing DIF items, the significant difference in practice scores remained virtually unchanged. The effect size remained very large (Cohen′s *d* = 3.03 after item removal vs. 2.93 in the full scale), indicating that the observed difference was robust to the removal of DIF items and unlikely to be explained solely by measurement artifacts. This finding suggests that the lower practice scores observed among physicians may reflect a genuine difference in self‐reported adherence to contact isolation precautions rather than an artifact of DIF.

Overall, these findings suggest that certain items in the isolation precautions questionnaire, particularly Item 8, may require correction or standardization when used in KAP studies. Although differences in questionnaire scores may be influenced by item functioning, the persistence of the practice‐score difference after removal of substantial DIF items suggests that this finding is not explained solely by measurement effects. After excluding items with significant DIF, nurses and physicians showed high knowledge scores, whereas practice scores remained notably low, particularly among physicians. This finding aligns with previous studies [16–18]. Nurses typically have more direct and frequent patient contact and are more involved in implementing contact isolation measures. In contrast, physicians may possess a broader understanding of infectious diseases and treatment options but may be less directly involved in the daily implementation of contact isolation protocols.

Many studies in this area have primarily compared mean differences among specific groups, such as nurses or medical students. For example, Santos et al. [21] focused solely on evaluating nursing undergraduate students’′ knowledge regarding contact precautions. Askarian et al. [16] compared mean scores of KAPknowledge, attitude, and practice among nurses, midwives, and lecturers. Another study by Askarian et al. [32] compared mean scores among medical students, including students, externs, and interns . Mousavi et al. [23](2021) identified substantial DIF between medical students and nursing groups using subscales of the standard isolation precaution questionnaire, emphasizing the need for cautious interpretation of results .

A major strength of this study is the use of DIF analysis, which provides a clearer understanding of differences between nurses and physicians. While Although disparities between these groups are frequently reported in the literature, our findings indicate that some observed differences may be related to measurement bias rather than true differences in the underlying constructs. For example, in the attitude domain, the significant difference disappeared after removing items with substantial DIF, whereas the difference in the practice domain persisted. These findings highlight the importance of assessing measurement equivalence when interpreting cross‐professional differences and developing training programs.

The observed domain score was used as the matching variable, and iterative purification was not performed because each domain contained only eight items and a high proportion exhibited DIF. Under these conditions, purification would have substantially reduced the number of available anchor items and may have produced an unstable matching variable. Instead, sensitivity analyses (Table 4) were conducted to evaluate the practical impact of DIF and provide additional evidence regarding the robustness of the observed between‐group comparisons.

## 5. Limitations

Few limitations are acknowledged in this study:

1. Although the unequal numbers of nurses and physicians reflected the workforce composition of the participating hospital, the smaller physician sample may have limited the precision and generalizability of the findings. Firth logistic regression was used to reduce potential estimation bias associated with unequal group sizes; the modest sample size of physicians may limit the generalizability of our findings to the broader physician population. In addition, because the study was conducted in a single tertiary‐care hospital, the findings may not be generalizable to other healthcare settings. Future multicenter studies including larger physician samples are warranted.
2. Dichotomization of the practice‐domain items from the original five‐point Likert scale followed the original scoring procedure and previous studies [16–18], reflecting a clinically meaningful compliance threshold. However, collapsing five response categories into a binary outcome reduced response variability and may have decreased the sensitivity to detect subtle DIF as well as the precision of between‐group score comparisons. Future studies using ordinal DIF methods that retain the full range of response categories may provide additional psychometric insight
3. As with most KAP studies, the data were based on self‐reported questionnaire responses and may therefore be subject to recall or social desirability bias, particularly in the practice domain.
4. The comparison of mean scores after excluding items with DIF should be interpreted as a sensitivity analysis rather than evidence for a revised scale structure. Because removal of items may reduce content coverage and alter the construct being measured, the shortened domains cannot be considered psychometrically equivalent to the original instrument without further validation. Internal consistency was re‐estimated for the reduced item sets (Cronbach′s alpha: knowledge = 0.631, attitude = 0.772, and practice = 0.617). The lower alpha observed in the modified practice domain may partly reflect the reduced number of items after DIF removal. Additional studies are needed to establish the reliability and validity of any modified version of the scale.

## 6. Conclusions

This study demonstrates that several items of the contact precautions questionnaire may be understood differently by nurses and physicians, suggesting that some items may not function similarly across these professional groups. Therefore, caution is warranted when comparing KAP scores between nurses and physicians. Items showing substantial and consistent DIF (e.g., Item 8 in the knowledge and attitude domains) may warrant careful review and revision to improve clarity and cross‐group comparability; if DIF persists after refinement, removal or alternative scoring approaches may be considered. Researchers are encouraged to evaluate measurement invariance before drawing conclusions from group comparisons. These findings apply specifically to the contact isolation precautions questionnaire examined in this study and to the nurses and physicians included in this study; the observed DIF patterns should not be assumed to apply to other infection prevention and control instruments or populations. Future studies should replicate these findings in multicenter samples and examine whether profession‐sensitive educational strategies are warranted once adequate cross‐group comparability is established.

## Author Contributions

N.K. contributed to data analysis, manuscript drafting, and study implementation. Z.S. contributed to the study conception, implementation of the study, design, analysis, interpretation, and critical involvement in drafting of the manuscript. P.J. was involved in interpretation of data, drafting and revising the manuscript. M.A. contributed to collection and interpretation of data. Each author contributed important intellectual content during manuscript drafting or revision and accepts accountability for the overall work by ensuring that questions pertaining to the accuracy or integrity of any portion of the work are appropriately investigated and resolved.

## Funding

This work was supported by Shiraz University of Medical Sciences, 10.13039/501100004320, 21402.

## Ethics Statement

The questionnaire data used in this study were originally collected in May 2019 as part of a larger approved research project at Namazi Hospital. The present study consisted of a secondary analysis of these existing data and received ethical approval from the Ethics Committee of Shiraz University of Medical Sciences (IR.SUMS.REC.1399.1036; approved on December 22, 2020).

## Consent

The authors have nothing to report.

## Conflicts of Interest

The authors declare no conflicts of interest.

## Supporting information


**Supporting Information** Additional supporting information can be found online in the Supporting Information section. Table S1: English translation of the original Persian questionnaire items used in the differential item functioning (DIF) analysis.

## Data Availability

The data that support the findings of this study are available on request from the corresponding author. The data are not publicly available due to privacy or ethical restrictions.

## References

[1] Allegranzi B. , Nejad S. B. , Combescure C. , Graafmans W. , Attar H. , Donaldson L. , and Pittet D. , Burden of Endemic health-care-associated Infection in Developing Countries: Systematic Review and Meta-Analysis, Lancet. (2011) 377, no. 9761, 228–241, 10.1016/S0140-6736(10)61458-4, 21146207. 21146207

[2] Allegranzi B. , Bagheri Nejad S. , Garcia Castillejos G. , Kilpatrick C. , Kelley E. , and Mathai E. , Report on the Burden of Endemic Health Care-Associated Infection Worldwide: A Systematic Review of the Literature, 2011, World Health Organization, http://apps.who.int/iris/bitstream/10665/80135/1/9789241501507_eng.pdf.

[3] Murphy F. , Tchetchik A. , and Furxhi I. , Reduction of Health Care-Associated Infections (HAIs) With Antimicrobial Inorganic Nanoparticles Incorporated in Medical Textiles: An Economic Assessment, Nanomaterials. (2020) 10, no. 5, 10.3390/nano10050999, 32456213. PMC7279532 32456213

[4] Mudedla S. , Tej W. L. , Reddy K. T. , and Sowribala M. , A Study on Knowledge and Awareness of Standard Precautions Among Health Care Workers at Nizam’s Institute of Medical Sciences Hyderabad, Journal of National Accreditation Board for Hospitals & Healthcare Providers. (2014) 1, no. 2, 34–38, 10.4103/2348-6139.151296.

[5] Vaz K. , McGrowder D. , Alexander-Lindo R. , Gordon L. , Brown P. , and Irving R. , Knowledge, Awareness and Compliance With Universal Precautions Among Health Care Workers at the University Hospital of the West Indies, Jamaica, International Journal of Occupational and Environmental Medicine. (2010) 1, no. 4, 171–181, 23022806. 23022806

[6] Bosques-Padilla F. J. , Vázquez-Elizondo G. , Villaseñor-Todd A. , Garza-González E. , Gonzalez-Gonzalez J. A. , and Maldonado-Garza H. J. , Hepatitis C Virus Infection in Health-Care Settings: Medical and Ethical Implications, Annals of Hepatology. (2010) 9, no. 2, S132–S140, 10.1016/S1665-2681(19)31738-7. 20714010

[7] Bianco A. , Bova F. , Nobile C. G. , Pileggi C. , Pavia M. , and Angelillo I. F. , Healthcare Workers and Prevention of Hepatitis C Virus Transmission: Exploring Knowledge, Attitudes and Evidence-Based Practices in Hemodialysis Units in Italy, BMC Infectious Diseases. (2013) 13, 76, 10.1186/1471-2334-13-76, 23391009. 23391009 PMC3571883

[8] Westermann C. , Peters C. , Lisiak B. , Lamberti M. , and Nienhaus A. , The Prevalence of Hepatitis C Among Healthcare Workers: A Systematic Review and Meta-Analysis, Occupational and Environmental Medicine. (2015) 72, no. 12, 880–888, 10.1136/oemed-2015-102879, 26438666. 26438666 PMC4680146

[9] Coppola N. , De Pascalis S. , Onorato L. , Calò F. , Sagnelli C. , and Sagnelli E. , Hepatitis B Virus and Hepatitis C Virus Infection in Healthcare Workers, World Journal of Hepatology. (2016) 8, no. 5, 273–281, 10.4254/wjh.v8.i5.273, 26925201. 26925201 PMC4757650

[10] Weiner L. M. , Webb A. K. , Limbago B. , Dudeck M. A. , Patel J. , Kallen A. J. , Edwards J. R. , and Sievert D. M. , Antimicrobial-Resistant Pathogens Associated With Healthcare-Associated Infections: Summary of Data Reported to the National Healthcare Safety Network at the Centers for Disease Control and Prevention, 2011–2014, Infection Control & Hospital Epidemiology. (2016) 37, no. 11, 1288–1301, 10.1017/ice.2016.174, 27573805. 27573805 PMC6857725

[11] Dhar S. , Marchaim D. , Tansek R. , Chopra T. , Yousuf A. , Bhargava A. , Martin E. T. , Talbot T. R. , Johnson L. E. , Hingwe A. , Zuckerman J. M. , Bono B. R. , Shuman E. K. , Poblete J. , Tran M. A. , Kulhanek G. , Thyagarajan R. , Nagappan V. , Herzke C. , Perl T. M. , and Kaye K. S. , Contact Precautions: More Is Not Necessarily Better, Infection Control & Hospital Epidemiology. (2014) 35, no. 3, 213–219, 10.1086/675294, 24521583. 24521583

[12] Garner J. S. and the Hospital Infection Control Practices Advisory Committee , Guideline for Isolation Precautions in Hospitals, Infection Control & Hospital Epidemiology. (1996) 17, no. 1, 54–80, 10.1017/S0195941700006123. 8789689

[13] Siegel J. D. , Rhinehart E. , Jackson M. , and Chiarello L. , 2007 Guideline for Isolation Precautions: Preventing Transmission of Infectious Agents in Healthcare Settings, American Journal of Infection Control. (2007) 35, no. 10, S65–S164, 10.1016/j.ajic.2007.10.007, 18068815. 18068815 PMC7119119

[14] Carroll C. , Apoorva S. , Camins B. , Woodward J. , and Jones M. , Healthcare Workers′ Knowledge and Beliefs on Contact Isolation Precautions, American Journal of Infection Control. (2005) 33, no. 5, e81–e82, 10.1016/j.ajic.2005.04.096.

[15] Coffman S. L. , Mutnick R. , and Herwaldt L. A. , Knowledge and Practice of HCW Regarding Clostridium difficile Infection and Contact Precautions in an Academic Medical Center, American Journal of Infection Control. (2011) 39, no. 5, 10.1016/j.ajic.2011.04.128.

[16] Askarian M. , Shiraly R. , and McLaws M.-L. , Knowledge, Attitudes, and Practices of Contact Precautions Among Iranian Nurses, American Journal of Infection Control. (2005) 33, no. 8, 486–488, 10.1016/j.ajic.2005.06.001, 16216666. 16216666

[17] Askarian M. , Mirzaei K. , Mundy L. M. , and McLaws M.-L. , Assessment of Knowledge, Attitudes, and Practices Regarding Isolation Precautions Among Iranian Healthcare Workers, Infection Control & Hospital Epidemiology. (2005) 26, no. 1, 105–108, 10.1086/502495, 15693417. 15693417

[18] Askarian M. , Shiraly R. , Aramesh K. , and McLaws M.-L. , Knowledge, Attitude, and Practices Regarding Contact Precautions Among Iranian Physicians, Infection Control & Hospital Epidemiology. (2006) 27, no. 8, 868–872, 10.1086/506411, 16874649. 16874649

[19] Walker C. M. , What′s the DIF? Why Differential Item Functioning Analyses Are an Important Part of Instrument Development and Validation, Journal of Psychoeducational Assessment. (2011) 29, no. 4, 364–376, 10.1177/0734282911406666.

[20] Martinková P. , Drabinová A. , Liaw Y.-L. , Sanders E. A. , McFarland J. L. , and Price R. M. , Checking Equity: Why Differential Item Functioning Analysis Should be a Routine Part of Developing Conceptual Assessments, CBE—Life Sciences Education. (2017) 16, no. 2, 10.1187/cbe.16-10-0307, 28572182. PMC5459266 28572182

[21] Santos J. S. , Corrêa I. , and Salgado M. E. , Knowledge of Nursing Undergraduate Students About the Use of Contact Precautions Measures, Investigación y Educación en Enfermería. (2014) 32, no. 3, 464–472, http://hdl.handle.net/11449/135321.

[22] Lee S. , Detecting Differential Item Functioning Using the Logistic Regression Procedure in Small Samples, Applied Psychological Measurement. (2017) 41, no. 1, 30–43, 10.1177/0146621616668015, 29881077. 29881077 PMC5978487

[23] Mousavi A. , Momeni M. , Danaei M. , and Askarian M. , A Differential Item Functioning (DIF) Study of the Infection Control Standard Isolation Precaution Instrument Across Gender and Major Among Healthcare Workers, Shiraz E-Medical Journal. (2021) 22, no. 12, 10.5812/semj.110977.

[24] Puhr R. , Heinze G. , Nold M. , Lusa L. , and Geroldinger A. , Firth′s Logistic Regression With Rare Events: Accurate Effect Estimates and Predictions?, Statistics in Medicine. (2017) 36, no. 14, 2302–2317, 10.1002/sim.7273, 28295456. 28295456

[25] Heinze G. , Ploner M. , Dunkler D. , and Southworth H. , logistf: Firth′s Bias-Reduced Logistic Regression (R package version 1.23) [Computer software], 2018, logistf, 10.32614/CRAN.package.logistf.

[26] Faghih M. , Bagheri Z. , Stevanovic D. , Ayatollahi S. M. T. , and Jafari P. , A Comparative Study of the Bias Correction Methods for Differential Item Functioning Analysis in Logistic Regression With Rare Events Data, BioMed Research International. (2020) 2020, no. 1, 1632350, 10.1155/2020/1632350, 32185193. 32185193 PMC7060847

[27] Stevanovic D. and Jafari P. , A Cross-Cultural Study to Assess Measurement Invariance of the KIDSCREEN-27 Questionnaire Across Serbian and Iranian Children and Adolescents, Quality of Life Research. (2015) 24, no. 1, 223–230, 10.1007/s11136-014-0754-0, 25034175. 25034175

[28] Jafari P. , Allahyari E. , Salarzadeh M. , and Bagheri Z. , Item-Level Informant Discrepancies Across Obese–Overweight Children and Their Parents on the PedsQL 4.0 Instrument: An Iterative Hybrid Ordinal Logistic Regression, Quality of Life Research. (2016) 25, no. 1, 25–33, 10.1007/s11136-015-1046-z. 26081294

[29] Jafari P. , Stevanovic D. , and Bagheri Z. , Cross-Cultural Measurement Equivalence of the KINDL Questionnaire for Quality of Life Assessment in Children and Adolescents, Child Psychiatry & Human Development. (2016) 47, no. 2, 291–304, 10.1007/s10578-015-0568-5, 26184967. 26184967

[30] Taber K. S. , The Use of Cronbach′s Alpha When Developing and Reporting Research Instruments in Science Education, Research in Science Education. (2018) 48, no. 6, 1273–1296, 10.1007/s11165-016-9602-2.

[31] Crane P. K. , Hart D. L. , Gibbons L. E. , and Cook K. F. , A 37-Item Shoulder Functional Status Item Pool Had Negligible Differential Item Functioning, Journal of Clinical Epidemiology. (2006) 59, no. 5, 478–484, 10.1016/j.jclinepi.2005.10.007, 16632136. 16632136

[32] Askarian M. , Aramesh K. , and Palenik C. J. , Knowledge, Attitude, and Practice Toward Contact Isolation Precautions Among Medical Students in Shiraz, Iran, American Journal of Infection Control. (2006) 34, no. 9, 593–596, 10.1016/j.ajic.2006.03.005, 17097455. 17097455

[33] Khoddami N. , Application of Weighted and Penalized Binary Logistic Regression for Rare Events to Evaluate Differential Item Functioning of Contact Isolation Precautions Questionnaire in Nurses and Physicians of Namazi Hospital, Shiraz, MSc Thesis. (2021) Shiraz University of Medical Sciences.

