# Supplementary material for: An Investigation of Differential Item Functioning in the Infection Control Contact Isolation Precaution Instrument Among Nurses and Physicians
Source: Biomed Res Int. 2026 Sep 16;2026:9324434. doi: 10.1155/bmri/9324434 (PMC13579533; doi:10.1155/bmri/9324434)
Supplement: Supplementary file 1 [file BMRI-2026-9324434-s001.docx]

**Supplementary Table S1.** English Translation of the Original Persian Questionnaire Items Used in the Differential Item Functioning (DIF) Analysis

| Item | Contact Precaution Items | knowledge | | | attitude | | | | | Practice | | | | | |
| --- | --- | --- | --- | --- | --- | --- | --- | --- | --- | --- | --- | --- | --- | --- | --- |
|  |  | Correct | Incorrect | I do not know | Very high | High | Moderate | Low | Unimportant | Always | Often | Sometimes | Rarely | Never |  |
| 1 | If a patient has a disease transmissible through contact, the patient should be admitted to a private room. |  |  |  |  |  |  |  |  |  |  |  |  |  |  |
| 2 | For a patient with a disease transmissible through contact, wearing gloves before entering the room and removing them before leaving the room is mandatory. |  |  |  |  |  |  |  |  |  |  |  |  |  |  |
| 3 | When providing healthcare services to a patient with a disease transmissible through contact, washing hands with disinfectant agents after removing gloves is mandatory. |  |  |  |  |  |  |  |  |  |  |  |  |  |  |
| 4 | For a patient with a disease transmissible through contact, if contact with the patient or the patient’s equipment is anticipated, wearing a gown upon entering the room and removing it upon leaving the room is necessary. |  |  |  |  |  |  |  |  |  |  |  |  |  |  |
| 5 | If a patient has a disease transmissible through contact, healthcare staff in the transport route and destination ward/unit (e.g., radiology) should be informed about the patient’s condition during patient transfer. |  |  |  |  |  |  |  |  |  |  |  |  |  |  |
| 6 | Noncritical patient-care equipment (e.g., a blood pressure device) used for a patient with a disease transmissible through contact should be dedicated exclusively to that patient. |  |  |  |  |  |  |  |  |  |  |  |  |  |  |
| 7 | Cleaning and disinfecting equipment that is commonly used for patients with diseases transmissible through contact is mandatory between contacts with those patients. |  |  |  |  |  |  |  |  |  |  |  |  |  |  |
| 8 | Simultaneous use of two plastic (freezer-type) gloves is equivalent to one surgical glove for patient care. |  |  |  |  |  |  |  |  |  |  |  |  |  |  |
